# Information and communication technology in personalized medicine: a clinical use-case for hepatocellular cancer

**DOI:** 10.1186/1878-5085-5-S1-A59

**Published:** 2014-02-11

**Authors:** Leonard Berliner, Heinz U Lemke, Eric van Sonnenberg, Hani Ashamalla, Malcolm D Mattes, David Dosik, Hesham Hazin, Syed Shah, Smruti Mohanty, Sid Verma, Giuseppe Esposito, Irene Bargellini

**Affiliations:** 1 New York Methodist Hospital, Brooklyn, NY; Weill Medical College of Cornell University, USA; 2 Technical University of Berlin; Berlin, Germany; 3 Kern/UCLA Medical Center, Bakersfield, CA, USA; 4 New York Methodist Hospital, Brooklyn, NY, USA; 5 Georgetown University Medical Center, Washington, DC, USA; 6 University Hospital Pisa, University of Pisa, Italy

## Scientific objectives

This study explores ways in which the requirements and interrelationships between Personalized Medicine (PM), clinical medical practice, and basic medical research could be best served by information and communication technologies (ICT). To avoid the problems inherent in formulating ICT solutions in isolation, a use-case was developed employing hepatocellular carcinoma (HCC). The subject matter was approached from four separate, but interrelated, tasks: (1) review of current understanding and clinical practices relating to HCC; (2) propose an ICT system for dealing with the vast amount of information relating to HCC, including clinical decision support and research needs; (3) determine the ways in which a clinical liver cancer center can contribute to this ICT approach; and, (4) examine the enhancements and impact that the first three tasks, and therefore Personalized Medicine, will have on the management of HCC. The development of an IT System for Personalized Healthcare (ITS-PHC) for HCC will provide a comprehensive system to identify and then determine the relative value of the wide number of variables or Information Entities (IEs): (1) factors reflecting clinical assessment of the patient including functional status, liver function, degree of cirrhosis, and comorbidities; (2) factors reflecting tumor biology, at a molecular, genetic and anatomic level; (3) factors reflecting tumor burden and individual patient response; and (4) factors reflecting medical and operative treatments and their outcomes.

## Technological approaches

It is our hypothesis, that if we can utilize patient-specific modeling techniques to generate valid Digital Patient Models (DPM) (utilizing these IEs) we may be able to develop a statistically valid methodology for predicting diseases, predicting treatment outcomes, preventing diseases or complications, and developing personalized treatment regimens. We are calling this proposed system Model-Based Medical Evidence (MBME), and as yet remains undeveloped. It is further postulated that Multi-Entity Bayesian Networks (MEBN) used in the construction of the DPM will be utilized in the development of a practical decision support system. Literature regarding HCC was analyzed, combining epidemiology; risk factors; infectious etiologies; pathology, microenvironment and biomarkers; screening and diagnostic technologies; treatment modalities. IEs, that will be used to populate the patient databases and MEBNs required for data mining and decision support, were identifed. This information was also used to reinforce a well-established treatment protocol, i.e. the Barcelona treatment algorithm, and, to add extensions that include enhanced screening and greater specifics regarding treatment selections.

## Results interpretation

New algorithms are presented that relate to alternatives in palliative treatments, down staging, and dealing with progression of disease. Treatments for HCC included in these algorithms include surgical resection, liver transplantation, percutaneous ablation, transarterial chemoembolization (TACE), local radiotherapy with Yttrium-90 microspheres, stereotactic body radiation therapy (SBRT), and systemic targeted therapy with the oral multikinase inhibitor, sorafenib. Currently, selection of the best single form of therapy or a combination of therapies, is unclear at this time, due insufficient available information. However, it is felt that as more patients are entered into the ITS-PHC, and as detailed Patient-Specific Models can be developed in sufficient numbers, it will be possible to assess and validate the treatment choices and criteria depicted in these algorithms in a manner that heretofore has not been achievable. Target benchmarks for the effective treatment of hepatocellular carcinoma can be established. Comparative studies of the costs of different treatment protocols may be evaluated with respect to successes and failures in treatment outcomes, and with respect to overall quality of care and the patient’s quality of life.

## Outlook and expert recommendations

We hope that by giving greater emphasis to individual patient characteristics and by extending the algorithms to a wider range of the patient treatment continuum (from screening to initial treatment to treatment of recurrences to hospice care) that we can improve the understanding, prevention, and treatment of HCC. We hope that through the use of enhanced ICT it may be possible to provide and validate a new methodology for Evidence-Based Medicine utilizing model theory, i.e. MBME. It is our assumption that the development of improved Information Technology will promote an approach based on a global understanding of disease and treatment outcomes, rather than reliance primarily upon local availability and expertise.

## Statement regarding authors

This abstract is based on a manuscript composed of multiple sections. Each author has made a substantial contribution.

